# Myositis Ossificans: A Mimicker of an Intramuscular Tumour

**DOI:** 10.5334/jbsr.3531

**Published:** 2024-02-14

**Authors:** Bjorn Valgaeren, Ione Limantoro

**Affiliations:** 1 Department of Radiology, University Hospitals Leuven, Herestraat 49, Leuven, 3000, Belgium; 2 Department of Radiology, University Hospitals Leuven, Herestraat 49, Leuven, 3000, Belgium

**Keywords:** Myositis ossificans, intramuscular tumour, tumour mimicker, do not touch lesion, MRI, ultrasound

## Abstract

*Teaching point:* Myositis ossificans is a do not touch lesion of which the radiological findings can be misleading in the early stages due to possible features mimicking a malignant process.

## Case

A 15-year-old boy presented with a painful swelling located above his knee, which was gradually increasing in size for the last 10 days without prior trauma. An ultrasound was performed at an outside institution, which suggested a malignant intramuscular mass. Subsequently, the patient was referred to a tertiary centre for further evaluation.

The paediatric oncologist ordered a new ultrasound and an urgent MRI.

**Figure 1:**
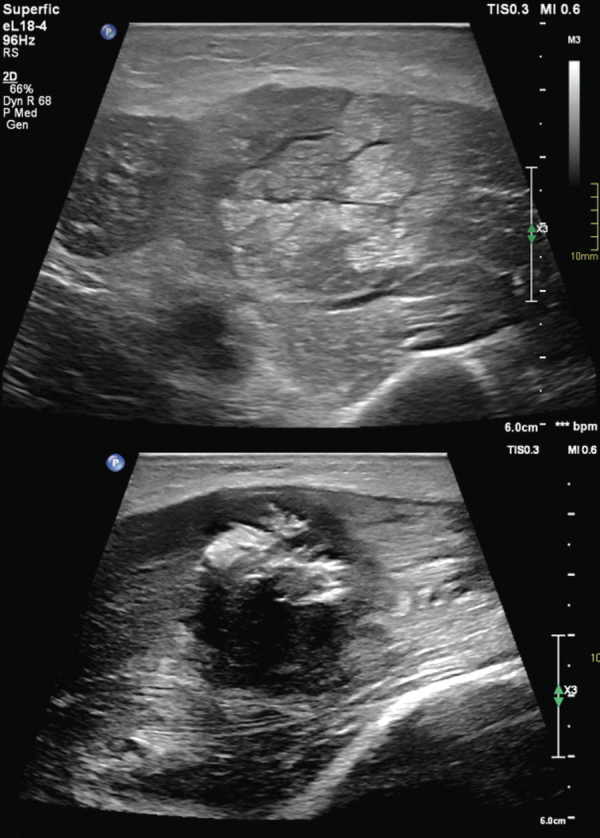
Ultrasound of myositis ossificans shows muscle oedema and calcifications.

This ultrasound confirmed the intramuscular mass, which was poorly defined, hypervascular, hypoechogenic and contained an incomplete ring of amorphous peripheral calcifications. A vast amount of intramuscular oedema was present surrounding the lesion, forming cable-like muscle fibres (Figure 1). These findings were suggestive of myositis ossificans.

**Figure 2:**
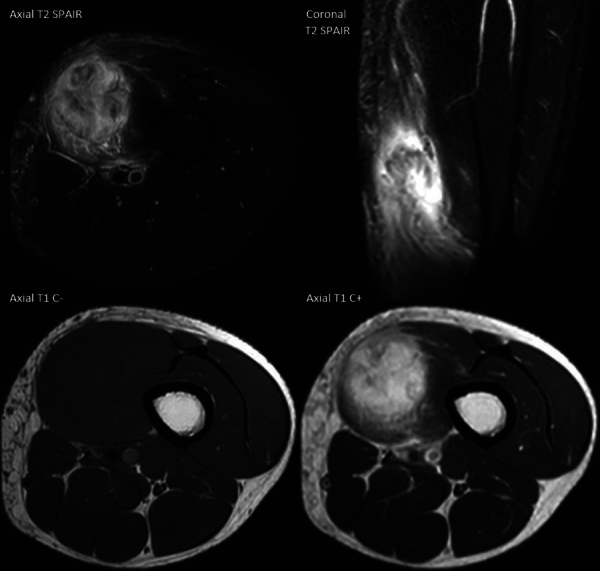
MRI of myositis ossificans shows marked muscle oedema and contrast enhancement.

The MRI showed a vividly enhancing, T1-hypointense and T2-hyperintense, lobulated mass in the distal vastus medialis oblique muscle without restricted diffusion. There was extensive oedema between the surrounding muscle fibres and in the subcutis. No extramuscular extension or bone involvement was seen (Figure 2).

**Figure 3:**
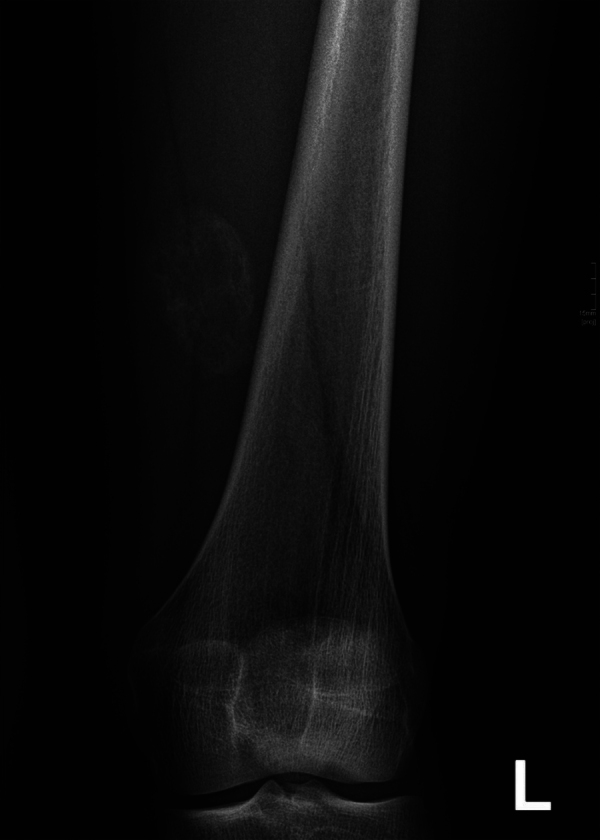
Radiograph of myositis ossificans shows progressive, oval, intramuscular ossification.

The diagnosis of myositis ossificans was suspected. A radiograph at this time showed no abnormalities. A follow-up radiograph 4 weeks later demonstrated peripheral calcifications, confirming the diagnosis (Figure 3).

## Comment

Myositis ossificans is a rare pseudotumoral lesion affecting the extraskeletal soft tissues, usually in the skeletal muscle of the extremities. This condition manifests itself usually after 4 to 12 weeks following blunt trauma, but may also be idiopathic. Young athletic males are generally affected [1].

Clinically, a painful swelling is seen, and joint stiffness may be present. This makes it difficult to differentiate from a malignant tumour [1].

The disease has three stages:

The early stage (<4 weeks) is characterised by an inflammatory reaction by tissue injury, facilitating proliferation of granulation tissue, and fibroblastic and osteoblastic cell proliferation. A large amount of oedema without apparent bone formation is seen on imaging [1].

In the intermediate stage (4–8 weeks), more immature lamellar bone starts to form, which can be seen more clearly on imaging as a ring of calcifications with less oedema [1].

Finally (>8 weeks), the bone starts to mature with almost no residual oedema present in the end stage [1].

The lesion might be mistaken for a sarcoma, particularly in the first and intermediate stages. The radiological key to the diagnosis is progressive peripheral bone formation, usually visible after 2 to 4 weeks on a radiograph (Figure 3), computed tomography (CT) or ultrasound.

When the diagnosis of myositis ossificans is unclear, one should wait 2 to 4 weeks for calcifications to develop. In the early stage, a biopsy is not helpful to exclude a malignant tumour, because the histological examination resembles sarcomas.
